# Prevalence and risk factors of anal human papillomavirus infection among HIV-negative men who have sex with men in Urumqi city of Xinjiang Uyghur Autonomous Region, China

**DOI:** 10.1371/journal.pone.0187928

**Published:** 2017-11-15

**Authors:** Tian Tian, Peierdun Mijiti, Huang Bingxue, Zhang Fadong, Abidan Ainiwaer, Sang Guoyao, Zhang Zhanlin, Yeledan Mahan, Tuo Xiaoqin, Gong Zheng, Dai Jianghong

**Affiliations:** 1 Department of Epidemiology and Biostatistics, School of Public Health, Xinjiang Medical University, Urumqi, Xinjiang, China; 2 Surgery Department of Toutunhe District General Hospital, Urumqi, Xinjiang, China; 3 Clinical Laboratory of Xinjiang Medical University First Affiliated Hospital, Urumqi, Xinjiang, China; Thai Red Cross AIDS Research Centre, THAILAND

## Abstract

**Background:**

Infection with human papillomavirus (HPV) is the most common sexually transmitted infection among men who have sex with men (MSM). Study on prevalence and risk factors of anal HPV infection among HIV-negative MSM in Northwestern China was rare.

**Methods:**

We performed a cross-sectional study of HPV prevalence using anal swab specimens among HIV-negative MSM in Urumqi city of Xinjiang Uyghur Autonomous Region, China between April 1^st^ and October 30^th^ in 2016. Prevalence of any anal HPV infection, high-risk and low-risk HPV infection was estimated. Risk factors associated with any anal HPV infection was analyzed using univariate and multivariate logistic regression models.

**Results:**

Among 538 potential participants, 500(92.9%) were recruited in this study. The genotyping results of anal HPV infection were available for all. Of them, 259 (51.8%), 190 (38.0%) and 141(28.2%) were positive for at least one of the targeted 37 HPV genotypes, high-risk HPV genotypes, and any low-risk HPV genotypes. The most prevalent anal HPV genotype was HPV 6(11.8%), followed by HPV 16(11.2%), HPV 11(10.8%), HPV 51(7.0%) and HPV 18(5.4%).Among those infected with at least one of the targeted 37 anal HPV genotypes, 75(29.0%), 155(59.8%) and 191(73.7%) were infected with 2-valent, quadrivalent and 9-valent HPV vaccine-covered genotypes. Receptive anal intercourse in the past year was the only predictor of any anal HPV infection in multivariate logistic regression model.

**Conclusion:**

Prevalence of any anal HPV infection and high-risk HPV infection among HIV-negative MSM in Urumqi city of Xinjiang is high. The majority of genotypes detected in our study were covered by quadrivalent and 9-valent HPV vaccines. Regular anal exams and early HPV vaccination among MSM may be considered in future HPV prevention programs in Xinjiang, China.

## Introduction

Human papillomavirus (HPV) is the most common sexually transmissible infectious agent worldwide [1]. So far, more than 200 types of HPV have been identified, and around 40 of these infect the squamous epithelium of the genital tract including the anus, cervix, vulva and vagina [2]. Importantly, most of HPV infections are transient and asymptomatic, since they are rapidly cleared by an intact immune system. However, in some instances, HPV may cause clinical manifestations of variable severity, spanning from benign warts (condylomata), mainly caused by the low-risk HPV 6 and 11, to cancerous lesions, caused by high-risk (HR) types (e.g. HPV 16 and 18) [3]. HR-HPV infection was thought to be responsible for 7–8% of all human malignancies [4] and was associated with majority of cervical cancers, anal cancers, vaginal cancers, vulvar cancers, and oropharyngeal carcinomas [5].

Recently, the interest on the burden of anal HPV infection has been growing because of increasing incidence of anal cancers among some at-risk population such as men who have sex with men (MSM) and HIV-infected individuals. Due to their sexual habits, MSM show a particularly high prevalence of HPV in the anal canal and develop lesions at this body site more frequently than any other population [6]. The prevalence of anal HPV infection among MSM was inconsistently reported in previous studies [7]. Prevalence of anal HPV infection among HIV-positive MSM in China was well documented [8–11]; however, data on prevalence of anal HPV among HIV-negative MSM, particularly in Northwestern China where reported number of MSM was increasing, was scarce. A better understanding of anal HPV infection among MSM may help inform strategies for the prevention of anal cancer in this population. In this study, we aimed to find out the prevalence and predictors of anal HPV infection among HIV-negative MSM in Urumqi city, China.

## Materials and methods

### Ethical statement

Xinjiang Medical University First Affiliated Hospital Ethics Committee reviewed and approved this study. Written informed consent obtained from each study participant before the interview and testing. All private information from questionnaire were kept confidential and used for population analysis only.

### Study population

We performed a cross-sectional study of HPV prevalence using anal swab specimens among HIV-negative MSM in Urumqi city of Xinjiang, China between April 1^st^ and October 30^th^ in 2016. Participants were recruited, through local non-government organization (Tianshan Volunteers Workstation), using the methods of website advertisement, WeChat group (Chinese social media mobile application software), MSM bars, sexually transmitted disease clinics, and voluntary HIV counseling and testing clinics. Additionally, those recruited were also encouraged to refer their peers to attend the study. Potential participants were invited to Tianshan Volunteers Workstation for questionnaire interview, HPV swabs, and HIV testing during the study period. Participants were eligible if they were male and at least 18 years old, self-reported having sex with men in the past year, were willing to provide anal swab specimens for HPV testing and blood for HIV testing, and were able to provide written informed consent. All participants were compensated 30 Chinese yuan (approximately four and half US dollar).

### Data collection

Data were collected using a questionnaire administered by trained interviewers who were also MSM in a private room. The questionnaire included information on socio-demographic characteristics (e.g. age group, ethnicity, education level, marital status, etc.), self-reported sexual orientation, heterosexual behavior in the past year, experience of anal intercourse in the past year, sexual role, commercial sex experience, circumcision status, STD infection in the past year, smoking status, and alcohol consumption.

### Specimen collection and laboratory testing

Human immunodeficiency virus infection status was determined by an enzyme immunoassay (Wantai Biological Pharmacy, Beijing, China) and was confirmed by HIV-1/2 Western blot assay (HIV Blot 2.2 WB; Genelabs Diagnostics, Singapore, Singapore).

Trained physician collected anal samples by inserting a saline water moistened nylon flocked swab 3–5cms in the anal canal and gently rotating it for about 2 minutes. The swab was then kept in 3 mL of sample transport medium for Hybribio 37 HPV GenoArray Diagnostic Kit (Hybribio Biotech Limited Corporation, Chaozhou, China). Hybribio 37 HPV GenoArray Diagnostic Kit Test is based on a flow-through hybridization and gene-chip method. The low-density gene chip was pre-fixed with 37 type-specific oligonucleotides and the genotype was analyzed using HybriMax (Hybribio Biotech Limited Corporation, Chaozhou, China).The final results were detected by colorimetric change on the membrane under direct visualization. Positive and negative controls were included in the GenoArray test kit in every PCR analysis as well as during the hybridization process for quality control. Mixtures of different specific probes can be used in the same well of a 42-well plate format allowing for multiplex analysis. Blue-purple spots were recognized as HPV positive. This testing kit can detect 37 common types of HPV, including 23 oncogenic or HR-HPV (16, 18, 26, 31, 33, 35,39, 45, 51, 52, 56, 58, 59, 66, 68, 82,83,53, 55, 34, 57, 69, and 71) and 14 non-oncogenic or LR-HPV (06, 11, 40, 42, 44, 54,61, 67, 70, 72, 73, 81, and 84). Study participants with genotyping positive for any HPV type were determined to be currently infected with HPV. The operating protocol has been described in detail elsewhere [12, 13].

### Statistical analysis

Questionnaires and laboratory results were entered using EpiData version 3.1(The Epi Data Association Odense, Denmark). Data were analyzed using SPSS version 17.0 (SPSS Inc., Chicago, IL, USA). Unconditional univariate logistic regression models were used to evaluate the association between each variable and any anal HPV infection. Multivariate logistic regression was applied to determine predictors of any anal HPV infection. All variables with *p*-values< 0.2 in univariate analyses were entered into the multivariate logistic model. Crude odds ratios (COR) and adjusted odds ratios (adjusted OR) were calculated, as appropriate along with 95% confidence intervals (CI).

## Results

### Characteristics of study participants

538 potential participants were invited for interview, HPV swabs and HIV testing. 16 did not give written consent, 12 did not provide blood for HIV testing, and eight did not show up at study site. Therefore, 502 individuals were recruited and two were positive for HIV. Finally, only data of 500 eligible HIV-negative participants were analyzed. Of them, 250 (50.0%) were aged≤ 29 years old, 277 (55.4%) were local residents, 457(91.4%) were of Han ethnicity, and 258(51.6%) had received education at the high school level or lower. In addition, 108 (21.6%) reported having sex with women in the past year, 439(87.8%) reported experience of anal intercourse in the past six months, 185 (37.0%) reported having been circumcised, only 44 (8.8%) reported having had STDs in the past year, and only 17 (3.4%) reported experience of commercial sex in the past year.

### Prevalence of HPV infection

The genotyping results of anal HPV were available for all participants (Table 1). Of them, 259 (51.8%) were positive for at least one of the targeted 37 HPV genotypes. All were negative for HPV 26, HPV 35, HPV 71, HPV 40 and HPV 67. Among those positive for any 37 HPV genotypes, 118(45.6%) were exclusively infected with HR-HPV, 69(26.6%) were exclusively infected with LR-HPV, and 72 (27.8%) were infected with both LR-HPV and HR-HPV. Among those exclusively infected with HR-HPV, 29.7% were multiple infections, while it was 13.0% among those with exclusively infected with LR-HPV.HPV 16 (11.2%) and HPV51 (7.0%) were found to be the most prevalent HR-HPV genotypes; HPV6 (11.8%) and HPV11 (10.8%) were the most prevalent LR-HPV genotypes. The prevalence of 2-valent, quadrivalent and 9-valent HPV vaccine genotypes among participants all tested for HPV were 15.0%, 31.0% and 38.2%, respectively. However, among those infected with at least one of the targeted 37 anal HPV genotypes, 75(29.0%), 155(59.8%) and 191(73.7%) were infected with 2-valent, quadrivalent and 9-valent HPV vaccine-covered genotypes.

**Table 1:** HPV detection and genotyping among 500 HIV-negative MSM.

HPV genotype	N	%
**Exclusively HR-HPV infection**	118	23.6
Single infection	83	70.3
Multiple infection	35	29.7
**Exclusively LR-HPV infection**	69	13.8
Single infection	60	87.0
Multiple infection	9	13.0
**HR-HPV/LR-HPV coinfection**	72	14.4
**HR-HPV types**		
HPV16	56	11.2
HPV51	35	7.0
HPV18	27	5.4
HPV58	25	5.0
HPV33	23	4.6
HPV 39	27	5.4
HPV 31	13	2.1
HPV 45	7	1.4
HPV 52	13	2.6
HPV 56	5	1.0
HPV 59	7	1.4
HPV 66	12	2.4
HPV 68	12	2.4
HPV 82	5	1.0
HPV 83	8	1.6
HPV 53	13	2.6
HPV 55	3	0.9
HPV 34	2	0.4
HPV 57	0	0
HPV 69	3	0.6
**LR-HPV types**		
HPV6	59	11.8
HPV11	54	10.8
HPV61	18	3.6
HPV84	14	2.8
HPV 42	1	0.2
HPV 54	9	1.8
HPV 70	6	1.2
HPV 73	6	1.2
HPV 44	1	0.2
HPV 72	1	0.2
HPV 81	9	1.8
**2-valent HPV vaccine types**	75	15.0
**Quadrivalent HPV vaccine types**	155	31.0
**9-valent HPV vaccine types**	191	38.2

Notes: HPV, human papillomavirus; MSM, men who have sex with men; HR-HPV, high risk HPV; LR-HPV, low risk HPV. 2-valent HPV vaccine types refer to HPV-16/18; Quadrivalent HPV vaccine types refer to HPV -6/11/16/18; 9-valent HPV vaccine types refer to HPV-6/11/16/18/31/33/45/52/58

### Risk factors associated with HPV infections

Univariate and multivariate logistic regression analyses were performed to assess the associations between variables and any HPV infection among HIV-negative MSM. Multivariate logistic regression analysis indicated that receptive anal intercourse in the past year was the only predictor of any anal HPV infection (Table 2 and Table 3).

**Table 2:** Characteristics of 500 study participants and univariate analysis of association between variables and any anal HPV infection [n (%)].

Characteristics	N	Any HPV infection		COR	*P* value
		yes	no		
Age group (years)					
≤29	250	129(49.8)	121(50.2)	0.87 (0.54–1.42)	0.575
30–39	161	81(50.3)	80(49.7)	0.83(0.49–1.39)	0.472
≥40	89	49(55.1)	40(44.9)	Ref.	
Ethnicity					
Han	457	231(50.5)	226(49.5)	Ref.	
Others	43	28(65.1)	15(34.9)	1.83(0.95–3.51)	0.068
Education level					
High school or lower	258	138(53.5)	120(46.5)	1.11(0.81–1.63)	0.435
University or higher	242	121(50.0)	121(50.0)	Ref.	
Marital status					
Unmarried	369	198(53.7)	171(46.3)	1.30 (0.64–2.63)	0.462
Married	97	45(46.4)	52(53.6)	0.97 (0.45–2.13)	0.947
Divorced/widowed	34	16(47.1)	18(52.9)	Ref.	
Self-reported sexual orientation					
Homosexual	383	197(51.4)	186(48.6)	0.94 (0.62–1.42)	0.768
Bisexual/heterosexual	117	62(53.0)	55(47.0)	Ref.	
Ever had sex with women in the past six month					
No	392	207(52.8)	185(47.2)	1.21 (0.79–1.85)	0.391
Yes	108	52(48.1)	56(51.9)	Ref.	
Number of male sexual partners in the past six months					
0	25	9(36.0)	16(64.0)	Ref.	
1–4	403	205(50.9)	198(40.1)	1.84(0.79–4.26)	0.154
≥ 5	72	45(62.5)	27(37.5)	2.96(1.15–7.63)	0.024
Frequency of condom use during homosexual behaviors in the past six months					
Never	44	22(50.0)	22(50.0)	1.01(0.54–1.91)	0.966
Sometimes	170	95(55.9)	75(44.1)	1.29(0.88–1.88)	0.198
Always	286	142(49.7)	144(50.3)	Ref	
Experience of anal intercourse in the past six months					
Yes	439	231(52.6)	208(47.4)	1.31(0.77–2.24)	0.325
No	61	28(45.9)	33(54.1)	Ref.	
Pattern of anal intercourse in the past year					
Mainly receptive	254	148(58.3)	106(41.7)	1.70(1.19–2.42)	0.003
Mainly insertive	246	111(45.1)	135(54.9)	Ref.	
Experience of commercial sex in the past year					
Yes	17	12(70.6)	5(29.4)	2.29 (0.80–6.61)	0.115
No	483	247(51.1)	236(48.9)	Ref.	
Circumcision					
Yes	185	101(54.5)	84(45.6)	1.19(0.82–1.79)	0.362
No	315	158(50.2)	157(49.8)	Ref.	
Ever had STDs in the past year					
Yes	44	25(56.8)	19(43.2)	1.25(0.67–2.33)	0.485
No	456	234(51.3)	22(48.7)	Ref.	
Smoking status					
Non-smokers	220	115(52.3)	105(47.7)	1.02(0.72–1.44)	0.851
Previous and current smokers	280	144(51.4)	136(48.6)	Ref.	
Alcohol consumption					
No	359	187(52.1)	172(47.9)	1.02 (0.52–1.30)	0.399
Yes	141	72(51.1)	69(48.9)	Ref.	

Notes: HPV, human papillomavirus; COR, crude odds ratio; STDs, sexual transmitted diseases.

**Table 3:** Multivariate analysis of risk factors for any HPV infection.

Variables	Adjusted OR		*P* value
Ethnic group			
Han	Ref.		
Others	1.84(0.95–3.56)		0.072
Experience of commercial sex in the past year			
Yes	2.46 (0.83–7.30)		0.104
No	Ref.		
Pattern of anal intercourse in the past year			
Mainly receptive	1.75 (1.22–2.51)		0.003
Mainly insertive	Ref.		
Number of male sexual partners in the past six month			
0	Ref.		
1–4	1.71(0.72–4.05)		0.222
≥ 5	2.10(0.84–5.27)		0.115
Frequency of condom use during homosexual behaviors in the past six month			
Never	1.10(0.57–2.12)		0.775
Sometimes	1.31(0.65–2.61)		0.452
Always	Ref.		

Notes: HPV, human papillomavirus; OR, odds ratio

## Discussion

In this study, we investigated the prevalence of anal HPV infection among HIV-negative MSM in Urumqi city of Xinjiang province. The prevalence of any anal HPV infection among HIV-negative MSM in our study was 51.8%, which was within the range of results reported by various studies in China (33.8%-62.8%) [10, 11, 13–14]. However, it was lower than studies conducted in other countries (70%-85.3%) [15–18]. The discrepancy between these studies might be due to different characteristics of target population (e.g. source of recruitment, whether community-based or clinics, demographic characteristics, behavioral factors) or the HPV testing methods. Hybrid Capture and polymerase chain reaction (PCR)-based assays (e.g. Linear Array, MY09/11, GP5+/6+, etc.) were widely used for HPV detection and genotyping in epidemiological studies [18]. FDA-approved Hybrid Capture 2 system can detect five LR-genotypes and 13 HR-genotypes, but it only distinguishes between the high-risk and low-risk groups and does not permit identification of specific HPV genotypes [19]. Additionally, the detection limit of approximately 5000 genome equivalents makes it less sensitive than PCR[20].The sensitivity and specificity of PCR-based assays may be varied due to choice of primers, size of PCR products, and methods of sequence-analysis of PCR products. In addition, the number of HPV genotypes detected by PCR-based assays is varied as well [21]. PCR followed by flow-through hybridization and gene chip technology was used in our study, and its sensitivity, specificity, and number of HPV genotypes detected (37 genotypes) were quite similar to the popular PCR-based Linear Array assay [22,23]. Therefore, comparison of anal HPV prevalence among MSM groups should be careful and cautious.

Approximately 90% of invasive anal cancers are attributed to single infections with a HR-HPV type [24]. Of HPV-positive anal cancers, the great majority are associated with HPV 16 type (85%), followed by HPV 18 (7%), with a combined HPV 16 and HPV 18 prevalence of 90% irrespective of geographic location [25].Therefore, screening for anal HR-HPV infection and HPV-related anal lesions may help to reduce the burden of anal cancer in this high risk population [26]. In our study, the prevalence of any HR-HPV type among HIV-negative MSM was 38.0%; this was higher than the results reported among HIV-negative MSM in Netherlands (33.6%), Thailand (36.6%) and China (27.1%) [14, 27, 28], but it was lower than the results of a study conducted in three cities of China (48.2%) [10]. Furthermore, the most prevalent HPV genotype in anal canal in our study was HPV 6(11.8%), followed by HPV 16(11.2%), HPV 11(10.8%), HPV 51(7.0%) and HPV 18(5.0%). The distribution of HPV genotype in anal canal was similar to that reported in other studies in China [10,14] and South Africa[15], but different from that reported in Thailand[29], in which the most common HPV types were HPV 16 (27%), HPV 58 (23%), HPV 51(18%), and HPV 39 (14%). These results implied HPV genotype distribution might be varied among different MSM groups, and thus understanding the distribution of HPV genotypes among local MSM may help to guide the introduction of proper HPV vaccine in the local MSM community.

It is noticeable that the great majority of HPV genotypes detected in our study were covered by both quadrivalent and 9-valent HPV vaccines. This implies a great number of HPV cases in MSM in Urumqi might be prevented by these two vaccines. However, until very recently 2-valent HPV vaccine became available in Chinese markets and it is the only available HPV vaccine in China. In addition, results from a previous study suggested very low awareness and acceptability of HPV vaccine among MSM in China, in which only 18.4% of MSM had heard of HPV, 10.2% had heard of HPV vaccine, and 20.2% were willing to take HPV vaccine before sexual debut [30]. Joint efforts should be made by MSM media and MSM community organizations and clinicians to enhance health education about HPV vaccination among MSM at proper age.

Risk factors associated with any anal HPV infection among MSM were various in previous studies. The most consistently reported risk factors for anal HPV infection were a past history of receptive anal intercourse, recent receptive anal intercourse and a higher number of male sexual partners in the past months [31–34]. Similarly, receptive anal intercourse in the past year was associated with anal HPV infection in our study. However, frequency of condom use during homosexual behaviors in the past six month and number of male sexual partners in the past six month were not associated with any anal HPV infection among MSM in our study. Results for these associations were not consistent in previous studies either [3, 34, 35–36]. Importantly, HPV transmission can occur through other non-intercourse anal sexual practices such as fingering, fisting and oral sex [37], this may partially explain why the association of frequency of condom use during homosexual behaviors with anal HPV infection was not observed in our study. Racial difference in anal HPV infection among MSM was observed in several studies [9, 31]. In our study, we found other ethnic minority groups were more likely to have anal HPV infection compared to Han ethnic group after adjustment of potential confounders, although this association was marginal (*p* = 0.072). This marginal racial disparity may be real or due to recruitment of a small number of ethnic minorities in our study. 25% of the total population of Urumqi city are ethnic minorities (2016 Statistical Yearbook of Xinjiang Uyghur Autonomous Region, China), and majority of them are Muslim. Recruitment of Muslim MSM participant in scientific studies is quite difficult because MSM of Muslim background often conceal their homosexual identity to avoid harsh stigma and discrimination by Islamic society [38, 39]. MSM with different religious belief and those irreligious may differ in homosexual behaviors, and religiosity may play an important role in transmission of infectious disease among MSM in China [40]. Future study should be done among Muslim MSM in Xinjiang region to investigate the real prevalence and risk factors of HPV infection among them.

The results in our study should be interpreted with caution. First, due to the nature of convenience sampling and source of recruitment, participants in our study may not represent all MSM in Urumqi city. This may lead to selection bias in our study. Second, cross-sectional detection of HPV may be transient deposition only, thus it is difficult to predict how many of the cases would persist. Third, we used interviewer-based questionnaire rather than self-administered questionnaire, this may introduce some information bias given the sensitivity of the topic (e.g. selective reporting of sexual behavior/habits due to prejudice/stigma, etc.). Fourth, HIV-positive MSM were not included in our study due to very low prevalence of HIV infection in our sample. Future HPV prevalence study should be focused on HIV-positive MSM in Xinjiang province.

## Conclusions

In conclusion, high prevalence of any anal HPV infection (51.8%) and any HR-HPV infection (38.0%) was observed among HIV-negative MSM in Urumqi city. The most prevalent HPV genotype was HPV 6, followed by HPV 16, HPV 11, HPV 51 and HPV 18, most of which were covered by quadrivalent and 9-valent HPV vaccine. Receptive anal intercourse in the past year was the only risk factor associated with any anal HPV infection in our study population. Regular anal exams and early HPV vaccination among MSM may be considered in future HPV prevention programs in Xinjiang, China.
